# *MicroRNA-101* targets von Hippel-Lindau tumor suppressor (*VHL*) to induce HIF1α mediated apoptosis and cell cycle arrest in normoxia condition

**DOI:** 10.1038/srep20489

**Published:** 2016-02-04

**Authors:** Ning Liu, Wu-Yan Xia, Shan-Shan Liu, Hai-Yan Chen, Lei Sun, Meng-Yao Liu, Lin-Feng Li, Hong-Min Lu, Yu-Jie Fu, Pei Wang, Hailong Wu, Jian-Xin Gao

**Affiliations:** 1 State Key Laboratory of Oncogenes and Related Genes, Renji-Med X Clinical Stem Cell Research Center, Ren Ji Hospital, School of Medicine, Shanghai Jiao Tong University, Shanghai, China

## Abstract

The activation/inactivation of HIF1α is precisely regulated in an oxygen-dependent manner. HIF1α is essential for hypoxia induced apoptosis and cell cycle arrest. Several recent studies indicated that the expression of miRNAs can be modulated by hypoxia. However, the involvement of miRNAs in the regulation of HIF1α induction remains elusive. In present study, we demonstrated that *miR-101* was rapidly and transiently induced after hypoxia in breast cancer cells. Over-expression of *miR-101* significantly inhibited cell proliferation in breast cancer cells through increased apoptosis and cell cycle arrest in normoxia condition. This inhibitory phenomenon seems due to *miR-101*-mediated induction of HIF1α, because we identified that *VHL*, a negative regulator of HIF1α, is a novel target of *miR-101* and over-expression of *miR-101* decreased VHL levels and subsequently stabilized HIF1α and induced its downstream target *VEGFA*. Furthermore, we demonstrated that siRNA-mediated knockdown of VHL or HIF1α overexpression could also induce apoptosis and cell cycle arrest whereas enforced expression of VHL, administration of anti-*miR-101* oligos or treatment of 2-MeOE2, an inhibitor of HIF1α, could rescue cells from such inhibition. These results reveal a novel regulatory mechanism of HIF1α induction in normoxia and suggest that *miR-101* mediated proliferation inhibition may through HIF1α mediated apoptosis and cell cycle arrest.

Hypoxia is one of the classical features in solid tumors. Hypoxia inducible factor-1 (HIF1), a heterodimer with HIF1α and HIF1β subunits, is the major activated transcriptional factor in response to hypoxia. Although HIF1β is constitutively expressed, the protein level of HIF1α is tightly regulated by the oxygen levels. When oxygen is adequate, HIF1α is hydroxylated by prolyl hydroxylase proteins (PHDs) and in turn recognized by the von Hippel-Lindau tumor suppressor, E3 ubiquitin protein ligase(VHL) for unbiquitin-mediated degradation^1^. Under hypoxia, the hydroxylase activity of PHDs is dramatically reduced; thus HIF1α is stabilized, dimerized with HIF1β to form HIF1 and translocated to the nucleus^2^. Because the increased protein level of HIF1α is frequently observed and correlated with poor prognosis in many cancer types, a long-lasting concept believes that HIF1α plays an oncogenic role in tumor growth^3^,^4^. However, some recent well-designed studies challenged this concept. Carmeliet *et al.* showed that HIF1α is required for hypoxia-mediated apoptosis and inhibition of cell proliferation^5^. HIF1α deficiency apparently inhibited tumor growth and enhanced tumor invasion in microenvironment with sufficient oxygen supply^6^. HIF1α stabilization due to loss of VHL decreased tumor growth^7^. Furthermore, HIF1α interacted with Myc or Cdc6 to induce cell cycle arrest in the absence of hypoxic signal^8^. Therefore, the role of HIF1α in tumor progression has been controversial.

MicroRNAs are evolutionally conserved short noncoding RNAs that negatively regulate the expression of both protein-coding and noncoding genes^9^,^10^. Due to the partial complement to their targets, a microRNA can regulate multiple genes’ expression simultaneously^11^. Recent studies have indicated that hypoxia modulates the expression of a specific set of microRNAs, termed “hypoxamirs”^12^,^13^,^14^,^15^. Given adaptation to hypoxia is essential for solid tumor progression, it’s intriguing to explore the feedback regulatory loops between “hypoxamirs” and hypoxic pathway. In this regard, several hypoxamirs have been shown to regulate hypoxic pathway in either negative or positive feedback loops. For example, by directly targeting HIF1α, two hypoxamirs, miR-20b and miR-199a, suppress hypoxia progression^16^,^17^. miR-424, induced by hypoxia, targets cullin 2 (CUL2) to stabilize HIF1α and enhance angiogenesis^18^.

*miR-101* is evolutionally conserved in vertebrates. Down-regulation of *miR-101* has been observed in various types of cancers, including prostate, colon, bladder, gastric, ovarian and breast cancers^19^,^20^,^21^,^22^,^23^,^24^,^25^. Ectopic expression of *miR-101* has been shown to induce cell apoptosis and inhibit cell proliferation^26^,^27^. However, our previous study demonstrated that enforced expression of *miR-101* confers cells estrogen-independent growth ability in breast cancer^28^. A recent report also showed that increased *miR-101* levels are negatively associated with the overall survival (OS) and disease-free interval (DFI) in patients with ovarian carcinoma and *miR-101* could target C-terminal binding protein-2 (CtBP2) to enhance the stemness of cancer cells^29^. These controversial results indicate that *miR-101* has divergent and even antagonistic roles in tumor progression. Interestingly, Kim JH *et al.* showed that hypoxia could induce *miR-101* expression in human umbilical vein endothelial cells (HUVECs), human brain microvascular endothelial cells (HBMECs), astrocytes, HeLa, and U937 cells^30^, suggesting that *miR-101* is a novel hypoxamir under the regulation of hypoxia. However, little is known about the roles of *miR-101* in regulating the components of hypoxia pathway.

In this study, we demonstrated that *miR-101* is an acute miRNA in response to hypoxia in breast cancer cells. *miR-101* could directly target *VHL*, a negative regulator of HIF1α, resulting in HIF1α induction in the absence of hypoxic signal. *miR-101* directed proliferation inhibition seems due to *HIF1*α mediated apoptosis and cell cycle arrest.

## Results

### Hypoxia induced *miR-101* expression in breast cancer cells

**Figure 1:**
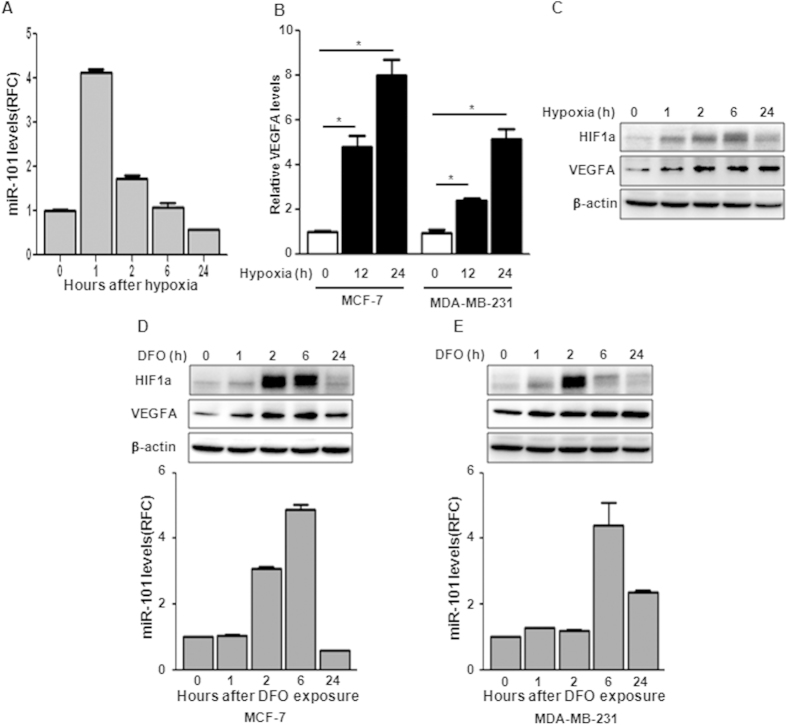
m*iR-101* is induced upon hypoxia exposure. (**A**) *miR-101* levels in MCF-7 after hypoxia exposure by qPCR. (**B**) VEGFA induction in MCF-7 and MDA-MB-231 cells by qPCR. (**C**) HIF1α and VEGFA induction after hypoxia by western blot. (**D**,**E**) HIF1α and VEGFA induction by western blot and *miR-101* expression by qPCR in MCF-7 (**D**) or MDA-MB-231 cells (**E**) after DFO treatment. Data were analyzed using Student’s t-test. *P < 0.05; **P < 0.01; ***P < 0.001.

Since Kim JH *et al.* showed induction of *miR-101* in Hela and U937 cells 12 h after hypoxia, we then examined whether hypoxia could induce *miR-101* expression in breast cancer cells. MCF-7 and MDA-MB-231 cells were exposed to hypoxia condition (1% Oxygen) and *miR-101* levels were measured by TaqMan qPCR at indicated time points. Figure 1A showed that *miR-101* expression rapidly reached a peak at 1 h after hypoxia exposure, and then declined to the basal level in 24 h in MCF-7 cells (Fig. 1A). We also observed similar kinetic changes but with much less induction of *miR-101* in MDA-MB-231 cells after hypoxia (data not shown). Consistent with previous reports, the expression of VEGFA, a well known downstream target of hypoxia, was dramatically induced after hypoxia (Fig. 1B). Western blot showed that the induction of HIF1α started in 1 h, reached its peak at 6 h and declined in 24 h after hypoxia (Fig. 1C). The induction of VEGFA was also confirmed by western blot (Fig. 1C). To further investigate the correlation between hypoxia and *miR-101*, we treated cells with the hypoxia-mimetic desferrioxamine (DFO). As shown in Fig. 1D,E, DFO treatment significantly increased HIF1α and VEGFA levels in both MCF-7 and MDA-MB-231 cells. Interestingly, unlike the rapid induction of *miR-101* in hypoxia treatment, miR-101 levels increased at 2 h and 6 h after DFO exposure in MCF-7 and MDA-MB-231 cells respectively (Fig. 1D,E).

### *miR-101* inhibits the cell proliferation in breast cancer cells

**Figure 2:**
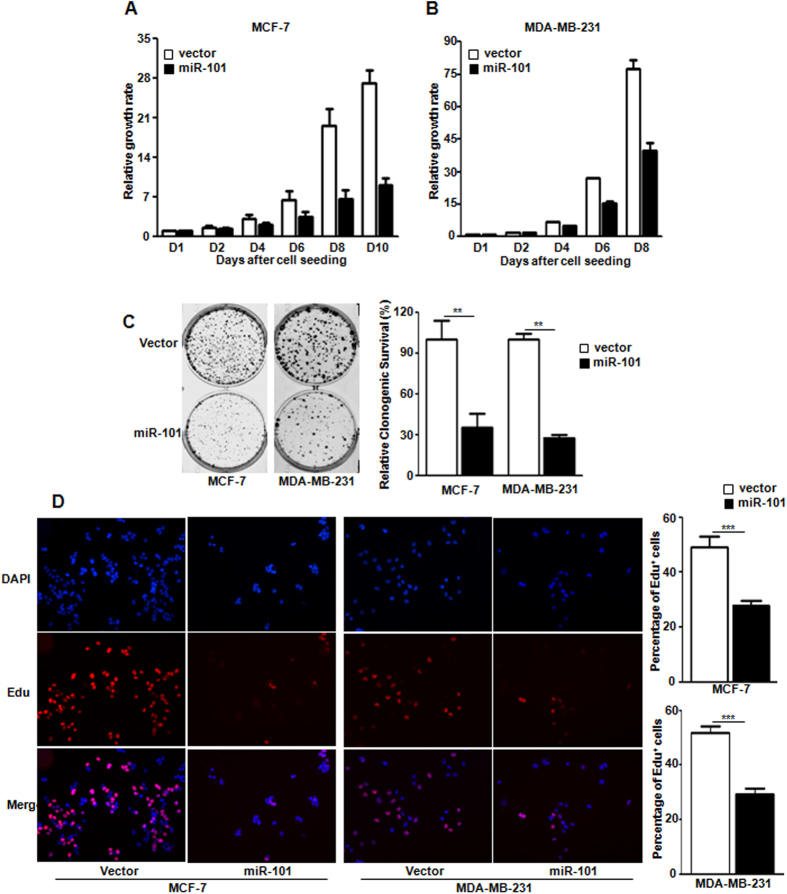
m*iR-101* inhibits cell proliferation. (**A,B**) Cell proliferation in MCF-7 (**A**) and MDA-MB-231 cells (**B**) with or without *miR-101* over-expression. (p < 0.0001 by two-way ANOVA). (**C**) Clonogenic survival in both MCF-7 and MDA-MBA-231 cells with or without *miR-101* over-expression. (**D**) Edu incorporation in MCF-7 and MDA-MB-231 cells with or without *miR-101* over-expression. Data were analyzed using Student’s t-test. *P < 0.05; **P < 0.01; ***P < 0.001.

To examine the functions of this novel hypoxamir in breast cancer cells, we stably expressed *miR-101* in MCF-7 and MDA-MB-231 cells after lentiviral infection. The ectopic expression of *miR-101* was confirmed by TaqMan qPCR in both cell lines (data not shown). Cell proliferation assay was performed. Figure 2A,B showed that over-expression of *miR-101* significantly inhibited the cell proliferation in both cell lines. To examine whether *miR-101* inhibits clonogenic survival, 2-dimension colony formation assay was performed. Compared to the control group, over-expression of *miR-101* significantly suppressed the colony formation to about 35% and 26% in MCF-7 and MDA-MB-231 cells respectively (Fig. 2C). To further assess whether *miR-101* suppresses cell proliferation, Edu incorporation was examined. Compared to the control group, *miR-101* expression dramatically impaired the incorporation of Edu in both MCF-7 and MDA-MB-231 cells (Fig. 2D).

### *miR-101* induces apoptosis and cell cycle arrest in breast cancer cells in normoxia

**Figure 3:**
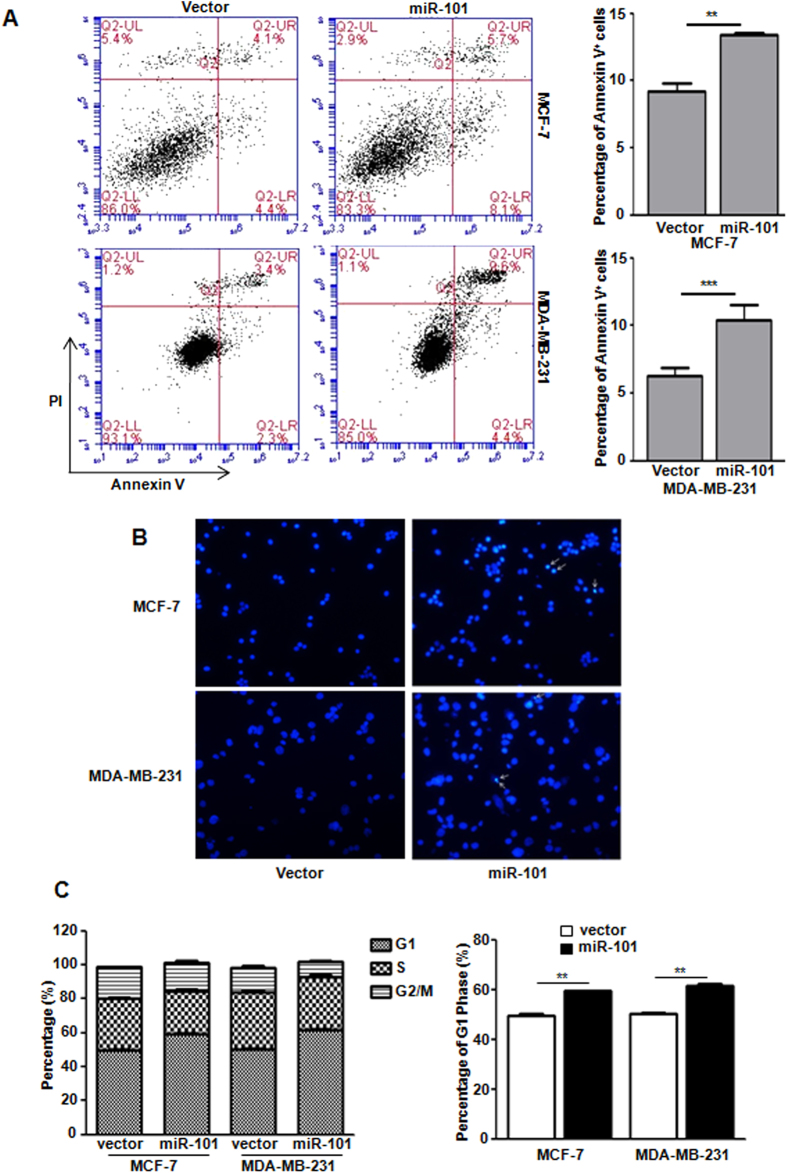
m*iR-101* induces apoptosis and cell cycle arrest. (**A**) Apoptosis assay in both MCF-7 and MDA-MB-231 cells with or without *miR-101* over-expression by Annexin V and PI double staining followed with flow cytometry analysis. (**B**) DAPI staining to detect apoptotic cells. (**C**) Cell cycle analysis in both MCF-7 and MDA-MB-231 cells with or without *miR-101* over-expression by PI staining followed by flow cytometry analysis. Data were analyzed using Student’s t-test. *P < 0.05; **P < 0.01; ***P < 0.001.

Since apoptosis and cell cycle arrest are tightly associated with reduced cell proliferation, we then determine whether *miR-101* could affect the apoptosis and cell cycle in breast cancer cells. Annexin V and Propidium iodide (PI) staining indicated that *miR-101* significantly induced cell apoptosis in both MCF-7 and MDA-MB-231 cells (Fig. 3A). DAPI staining further confirmed the *miR-101*-induced apoptosis in both cell lines (Fig. 3B). PI/FACS analysis demonstrated that *miR-101* over-expression caused significant G1-phase arrest in both cell lines (Fig. 3C). In addition, we examined the effects of *miR-101* on apoptosis and cell cycle of MCF-7 cells in hypoxia condition, but no significant changes were observed (Fig. S1). Given the essential role of CyclinD1 in regulating cell cycle, we started to check if *miR-101* mediated cell cycle arrest is due to the changes of CyclinD1. qPCR and western blot showed that neither mRNA nor protein levels of CyclinD1 was affected by *miR-101* in either normoxia or hypoxia condition (Fig. S2).

### VHL is a direct target of miR-101

**Figure 4:**
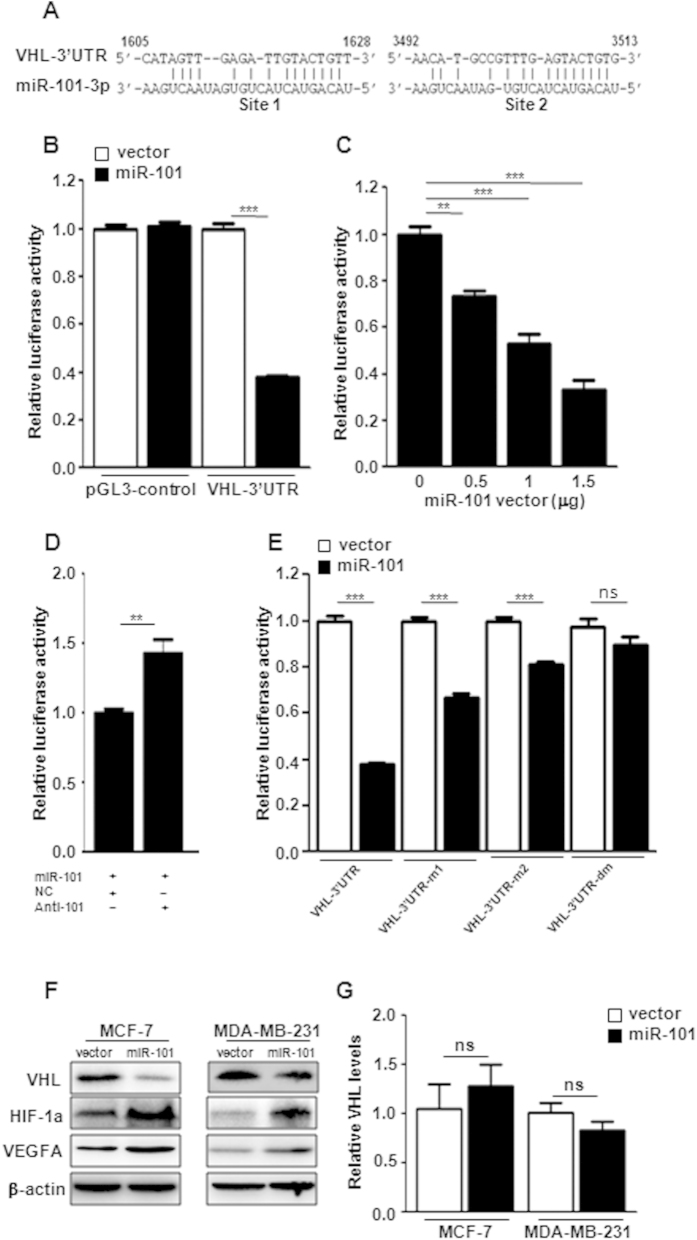
*VHL* is a direct target of *miR-101*. (**A**) Two putative binding sites of *miR-101* on the 3′UTR of *VHL*. (**B**) Suppression of the luciferase activity by miR-101. (**C**) Suppression of the luciferase activity by miR-101 in a dose dependent manner. (**D**) anti-miR-101 increased the luciferase activity in cells with miR-101 over-expression. (**E**) Effect of miR-101 on the luciferase activity of VHL-3′UTR, VHL-3′UTR-m1, VHL-3′UTR-m2 and VHL-3′UTR-dm. (**F**) HIF1α, VEGFA, VHL level changes in MCF-7 and MDA-MB-231 cells with or without *miR-101* over-expression by western blot. (**G**) mRNA level changes of VHL in MCF-7 and MDA-MB-231 cells with or without *miR-101* over-expression by qPCR. Data were analyzed using Student’s t-test. *P < 0.05; **P < 0.01; ***P < 0.001.

Previous studies have demonstrated that *miR-101* targets multiple genes, such as EZH2, COX-2, STMN1, ROCK2 and so on, to inhibit tumor growth^19^,^20^,^31^,^32^. In this study, we focused on exploring the putative regulation loops between *miR-101* and hypoxia pathway. After miRNA database search, we identified that VHL, a negative regulator of HIF1α, is a putative target of *miR-101* (miRDB v4.0). As shown in Fig. 4A, the VHL-3′UTR carries two putative binding sites of *miR-101*. We constructed a luciferase reporter vector carrying a *VHL* 3′UTR region containing those two binding sites. Compared to vector control, over-expression of *miR-101* significantly inhibited luciferase activity of VHL-3′UTR down to 40% as well as had no effect on the luciferase activity of pGL3-control vector (Fig. 4B). Figure 4C showed that *miR-101* mediated reduction of luciferase activity was in a dose-dependent manner. To assess the specificity of *miR-101* mediated suppression, we employed anti-*miR-101* oligos to block the suppression of *miR-101*. Compared to the negative control oligo, introduction of the anti-*miR-101* reversed the *miR-101*-mediated suppression of luciferase activity (Fig. 4D). To further confirm whether *miR-101* mediated suppression of luciferase activity is attributed to the direct binding of *miR-101* to the VHL-3′UTR, we mutated those two putative binding sites individually or both. *miR-101* induced suppression was significantly attenuated in VHL-3′UTR vectors with either site 1 or site 2 mutation and was completely abolished in VHL-3′UTR with site1 and site2 double mutations (Fig. 4E). These results indicated that VHL is a direct target of *miR-101*. To examine if *miR-101* also targets on other negative regulators of hypoxia pathway, such as PHD1, PHD2, PHD3 and PHD4, we did an extensive search for putative *miR-101* targets by employing a prediction program with less stringency (FINDTAR3: http://bio.sz.tsinghua.edu.cn/). FINDTAR3 indicated putative bindings between *miR-101* and all those four negative regulators (Fig. S3). However, luciferase reporter assay failed to validate those interactions (Fig. S3). To determine whether *miR-101* could directly downregulate endogenous VHL expression, we performed western blot in MCF-7 and MDA-MB-231 cells with stable ectopic expression of *miR-101*. As shown in Fig. 4F, *miR-101* significantly suppressed VHL protein levels in both cell lines and this suppression was through post-transcriptional regulation because *miR-101* over-expression did not reduce VHL mRNA levels (Fig. 4G). In response to VHL downregulation, the expression of HIF1α and VEGFA was increased (Fig. 4F).

### Down-regulation of VHL induces increased apoptosis and cell cycle arrest

**Figure 5:**
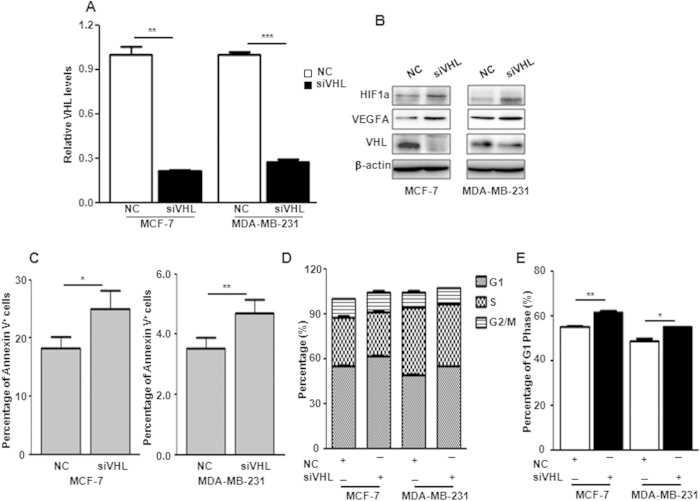
Knockdown of *VHL* induces apoptosis and cell cycle arrest. (**A**) VHL levels in MCF-7 and MDA-MB-231 cells transfected with siVHL by qPCR. (**B**) Protein level changes of VHL, HIF1α and VEGFA in MCF-7 and MDA-MB-231 cells transfected with siVHL by western blot. (**C–E**) Percentage of Annexin V positive cells (**C**), G1, S and G2/M phages (**D**) and G1 phase (**E**) in MCF-7 and MDA-MB-231 cells transfected with siVHL. Data were analyzed using Student’s t-test. *P < 0.05; **P < 0.01; ***P < 0.001.

To answer whether *miR-101*-mediated apoptosis and cell cycle arrest are due to *VHL* suppression, we employed *VHL* siRNA to specifically knockdown *VHL* expression. Realtime PCR and western blot validated successful knockdown of *VHL* in both mRNA and protein levels respectively in MCF-7 and MDA-MB-231 cells 48 hours after siVHL transfection (Fig. 5A,B). The consequent increase of HIF1α and VEGFA was also detected in cells with VHL suppression (Fig. 5B). Apoptosis and cell cycle assays were performed 48 hours post siVHL transfection. As shown in Fig. 5C–E, downregulation of VHL induced significant apoptosis and G1-phase arrest in MCF-7 and MDA-MB-231 cells compared to the negative control oligo.

### Overexpression of *VHL* rescues cells from *miR-101*-mediated apoptosis and cell cycle arrest

**Figure 6:**
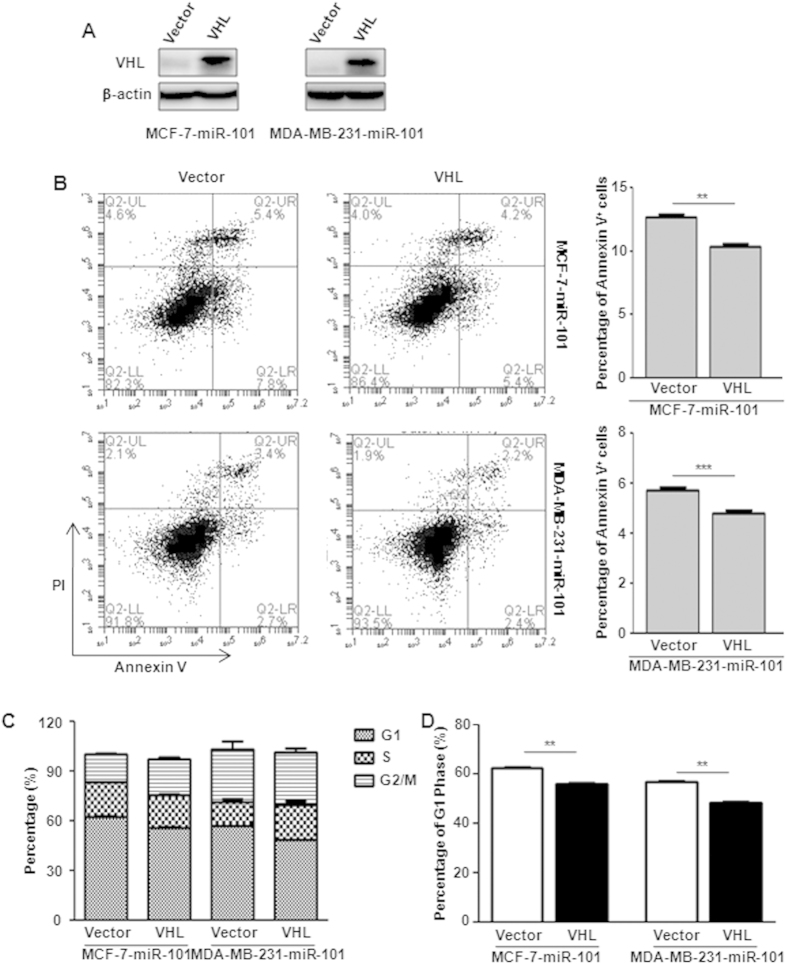
*VHL* restoration rescues cells from *miR-101*-mediated apoptosis and cell cycle arrest. (**A**) Ectopic expression of VHL in MCF-7-miR-101 and MDA-MB-231-miR-101 cells by western blot. (**B**) Apoptosis assay in both MCF-7-miR-101 and MDA-MB-231-miR-101 cells with or without *VHL* over-expression by Annexin V and PI double staining followed with flow cytometry analysis. (**C,D**) Percentage of G1, S and G2/M phages (**C**) and G1 phase (**D**) in MCF-7-miR-101 and MDA-MB-231-miR-101 cells with or without *VHL* over-expression. Data were analyzed using Student’s t-test. *P < 0.05; **P < 0.01; ***P < 0.001.

To further test whether miR-101 functions upstream of VHL to induce cell apoptosis and G1-phase arrest, we over-expressed *VHL* in both cell lines with stable ectopic *miR-101* expression (Fig. 6A). Annexin V and PI staining showed that *VHL* over-expression significantly blocked *miR-101* induced apoptosis in both cell lines (Fig. 6B). FACS assay indicated that miR-101 induced G1-phase arrest was significantly released upon VHL over-expression (Fig. 6C,D).

### Anti-miR-101 oligos block miR-101 mediated apoptosis and cell cycle arrest

**Figure 7:**
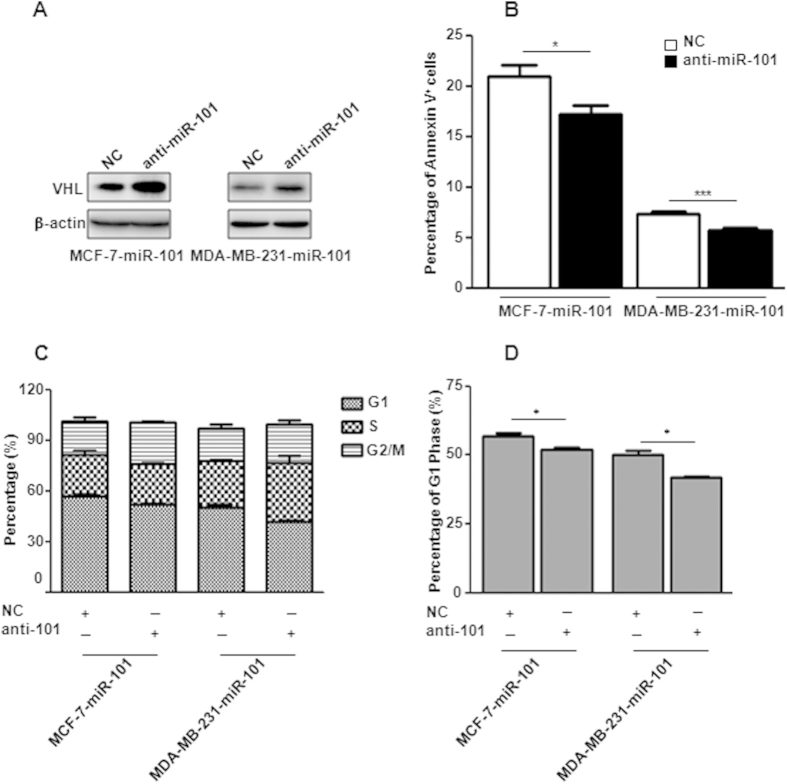
Blocking of *miR-101* releases cells from apoptosis and cell cycle arrest. (**A**) VHL level changes in MCF-7-miR-101 and MDA-MB-231-miR-101 cells transfected with anti-miR-101 oligos by western blot. (**B**) Percentage of Annexin V positive cells in MCF-7-miR-101 and MDA-MB-231-miR-101 cells transfected with anti-miR-101 oligos. (**C**,**D**) Percentage of G1, S and G2/M phages (**C**) and G1 phase (**D**) in MCF-7-miR-101 and MDA-MB-231-miR-101 cells. Data were analyzed using Student’s t-test. *P < 0.05; **P < 0.01; ***P < 0.001.

To answer whether inhibition of *miR-101* could rescue cells from *miR-101* induced apoptosis and cell cycle arrest, we transiently administrated anti-*miR-101* oligos into cells with stable *miR-101* over-expression. Compared with the negative control, released VHL repression was observed in cells transfected with anti-*miR-101* (Fig. 7A). Cell aopotosis and FACS assays showed that blocking of *miR-101* significantly rescued cells from *miR-101* induced apoptosis and G1 phase arrest in both breast cancer cell lines (Fig. 7B–D).

### HIF1a is required for miR-101 mediated apoptosis and cell cycle arrest

**Figure 8:**
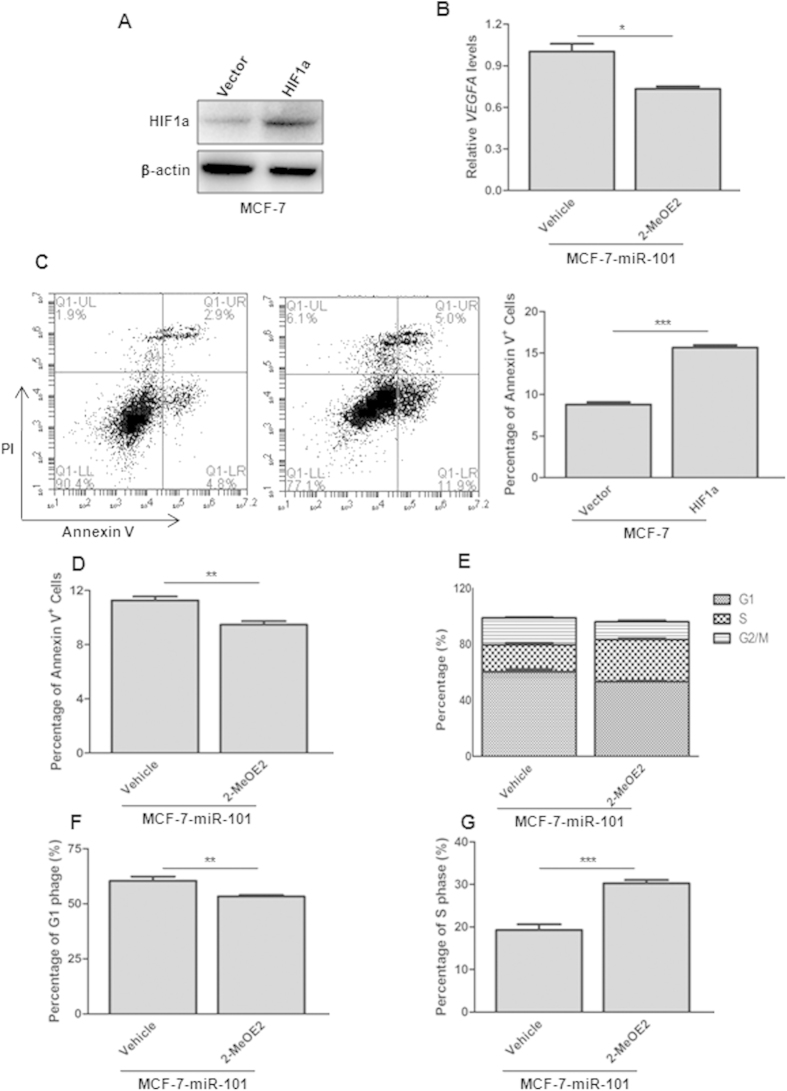
HIF1α is attributed to *miR-101* mediated apoptosis and cell cycle arrest. (**A**) HIF1a level change in MCF-7 cells infected with HIF1a expression vector by western blot. (**B**) VEGFA level change in MCF-7-miR-101 cells after 48 h 2-MeOE2 treatment by qPCR. (**C**) Apoptosis assay in MCF-7 cells with HIF1a over-expression by Annexin V and PI double staining followed with flow cytometry analysis. (**D**) Apoptosis assay in MCF-7-miR-101 cells after treated with 2-MeOE2. (**E–G**) Percentage of G1, S and G2/M phages (**E**), G1 phase (**F**) and S phase (**G**) in MCF-7-miR-101 cells after 2-MeOE2 treatment. Data were analyzed using Student’s t-test. *P < 0.05; **P < 0.01; ***P < 0.001.

To examine whether HIF1a over-expression could induce apoptosis, MCF-7 cells with stable HIF1a over-expression were generated after lentiviral infection. Ectopic HIF1a expression was confirmed by western blot (Fig. 8A). Annexin V and PI staining indicated that HIF1a over-expression increased the percentage of apoptosis cells to near 2-fold (Fig. 8C). To determine if HIF1a is required for miR-101 induced apoptosis and cell cycle arrest, we employed 2-MeOE, an inhibitor of HIF1a, to treat MCF-7 cells with stable miR-101 over-expression. 2-MeOE2 successfully inhibited HIF1a activity as the VEGFA level was significantly decreased upon 500nM 2-MeOE2 treatment for 48h (Fig. 8B). As shown in Fig. 8D–F, inhibition of HIF1α significantly rescued cells from *miR-101* mediated apoptosis and G1 phase arrest. Interestingly, consistent with a previous report that loss of HIF1α promotes G1 to S phase transition, MCF-7-miR-101 also showed significantly increased G1 to S progression upon 2-MeOE2 treatment (Fig. 8G).

## Discussion

In present study, we demonstrated that *miR-101* is a novel hypoxamir in breast cancer cells, whose expression was rapidly and transciently induced in 1 hour after hypoxia. Through directing cell apoptosis and cell cycle arrest, ectopic expression of *miR-101* suppressed proliferation in breast cancer cells. To our knowledge, we for the first time identified that *VHL* is a direct target of *miR-101* and demonstrated that *miR-101* could increase HIF1α protein levels by repressing *VHL* in normoxia condition. Furthermore, we showed that an increase of HIF1a levels by either direct HIF1a over-expression or by indirect knockdown of *VHL,* a negative regulator of HIF1a, can induce apoptosis and cell cycle arrest. In contrast, HIF1a downregulation by VHL over-expression, anti-*miR-101 oligo* administration or 2-MeOE2 treatment rescued cells from *miR-101*-mediated apoptosis and cell cycle arrest in breast cancer cells.

Hypoxic microenvironment commonly exists in many solid tumors, and is tightly associated with poor prognosis and resistance to therapy. HIF1α expression is induced in cells under hypoxia exposure and maintained at a basal level under normoxia^4^. Either the normoxic silencing or the hypoxic induction of HIF1α is orchestratedly modulated by multiple negative or positive regulators^33^. Hypoxia induced HIF1α dimerizes with HIF1β to transcript the expression of hundreds of downstream genes. miRNAs, a class of novel negative regulators of gene expression, have emerged in the downstream targets of HIF1α under hypoxia. These hypoxia mediated miRNAs are termed hypoxamiRs, including miR-210, miR-10b, miR-155, miR-372/373 and miR-424 whose expression was up-regulated, and miR-199a, miR-20b and miR-200b whose expression was down-regulated under hypoxia^16^,^17^,^18^,^34^,^35^,^36^,^37^,^38^,^39^,^40^,^41^. Interestingly, some of those hypoxamiRs, such as miR-20b, miR-199a and miR-424, can form negative or positive feedback loops to modulate hypoxia pathway^16^,^17^,^18^.

*miR-101*, a conserved miRNA, was first reported as a tumor suppressor in prostate cancer. Thereafter, accumulating evidence had demonstrated the tumor suppression feature of miR-101 in many other tumor types including colon cancer, hepatocellular carcinoma, bladder cancer, gastric cancer, non-small cell lung cancer, breast cancer, pancreatic cancer and esophageal cancer^20^,^21^,^22^,^25^,^26^,^42^,^43^,^44^. However, by adopting an unbiased miRNA library screening system, we previously demonstrated that over-expression of *miR-101* conferred cells estrogen-independent growth ability in MCF-7 and T47D cells, suggesting the oncogenic potential of *miR-101* in breast cancer^28^. Interestingly, the oncogenic role of *miR-101* has been recently reported by Dr. Weiping Zou’s group in ovarian cancer^29^. They demonstrated that Myeloid-derived suppressor cells (MDSCs) in ovarian carcinoma could induce the expression of *miR-101* and in turn enhance the stemness of cancer cells^29^. Thus, these controversial data indicate that *miR-101* plays a dual role as either a tumor suppressor or an oncogene in tumor progression. In present study, we demonstrated that *miR-101* was induced at the very early stage of hypoxia and *miR-101* could target VHL, resulting in stabilization of HIF1α in normoxia condition.

Emerging evidence has indicated that HIF1a has differential roles in tumor progression greatly dependent on the microenvironment of the tumor^6^. Blouw found that HIF1α deficient astrocytomas showed greater proliferation and invasion abilities when inoculated into the brain their natural habitat (with sufficient oxygen supplement) instead of the subcutaneous habitat (a poorly vascularized environment)^6^, suggesting the tumor suppressive role of HIF1a in the tumor with adequate oxygen supply. In gliomas with mutations in isocitrate dehydrogenase 1/2 (IDH1)IIDH2, HIF1α also presents as a tumor suppressor^45^. Koivunen found that the most common IDH mutant IDHR132H can activate PDHs to suppress HIF1α level, in turn promoting the proliferation and anchorage- independent growth abilities of immortalized astrocytes^45^. Therefore, HIF1α plays a suppressive role when the microenvironment of tumor is oxygen sufficient. Consistent with these previous findings, we demonstrated that miR-101 can target *VHL*, an inhibitor of HIF1α, to increase HIF1α level and in turn suppress cell proliferation via HIF1α mediated apoptosis and cell cycle arrest. Our finding suggested that the miR-101/VHL/HIF1α axis may play a tumor suppressive role when adequate oxygen was supplied to the tumor.

A recent paper reported that HIF1α plays a positive role in the proliferation in MDA-MB-231 cells^46^, which is opposite to our findings. However, it’s not convincing that Shi just employed a simple approach, siRNA mediated HIF1a knockdown, in their study to address the positive role of HIF1α. It’s also inappropriate for Shi to use only one siRNA duplex against HIF1a, because the phenotypes in Shi’s study may be probably caused by off-targeting.

In fact, several previous studies had also indicated the connection between *miR-101* and hypoxia pathway. For example, *miR-101* induction has been observed in HUVECs, HBMECs, astrocytes, HeLa, and U937 cells under hypoxia treatment^30^; inhibition of *miR-101* could rescue cardiomyocytes from apoptosis induced by hypoxia/reoxygenation^47^. Thus, previous data and the present study strongly implicated that *miR-101* mediated inhibition on cell proliferation in breast cancer cells seems due to HIF1α-induced apoptosis and cell cycle arrest. Recently, *miR-101* based targeted therapy had been reported in treatment of hepatocellular carcinoma in a mouse model with a great success^32^. However, when we appreciate the great potential of *miR-101* in tumor therapy, necessary cautions must be emphasized given the dual role of *miR-101* in tumor progression.

## Methods

### Cell Culture

HEK293T and human breast cancer cell lines MDA-MB-231 and MCF-7 were obtained from the American Type Culture Collection (ATCC, Manassas, VA, USA). All cell lines were maintained in DMEM medium (Gibco) supplemented with 10% fetal bovine serum (Gibco) and 0.1 mg/ml Penicillin-Streptomycin (Gibco) and incubated at 37 °C in a humidified chamber supplemented with 5% CO_2_.

### Hypoxia exposure

Cells were maintained in a hypoxic chamber flushed with a gas mixture containing 94%N_2_ and 5%CO_2_. Under these conditions, O_2_ levels in the medium were determined to be 1%. For DFO treatment, cells were treated with DFO (500 uM). Protein and RNA were isolated at indicated time points.

### Plasmid construction and transfection

HIFα over-expression vector was purchased from Addgene (Plasmid# 19365). To generate miR-101 expression vectors, a 420 bp fragment carrying pre-miR-101 was amplified from the MCF-7 genomic DNA by the high fidelity polymerase Phusion enzyme (New England Biolabs, Ipswich, MA) using PCR primers:

5′ tctagaTATTTCAGCCTCACCACTTGCT

5′ tctagaCCCCATGTTACAAAACAAGGCA.

The amplified fragment was cloned into the pLVX-IRES-ZsGreen vector (Clontech) at the Xba1 site.

To generate the luciferase reporter vector carrying the VHL-3′UTR region carrying the two putative binding sites of miR-101, we amplified a 2243 bp VHL-3′UTR region from the genome DNA of MCF-7 by the high fidelity polymerase Phusion enzyme (New England Biolabs, Ipswich, MA) using PCR primers:

5′ tctagaGGAGTAGCCTGGACTGTTTCAT

5′ tctagaTCCTTGGACAACACCAAAAACAC

The amplified fragment was cloned into the pGL3-control vector (Promega) at the Xba1 site.

To generate the VHL-3′UTR reporter vectors with mutated *miR-101* binding sites, we used *Phusion Site*-*Directed Mutagenesis* Kit (Life Technologies) to directly mutate those two binding sites by using primers:

VHL-UTR-mut1f: p-catagttgagattCACACACtcatacagtttta

VHL-UTR-mut1r: p-taaaaaccaaccaaaatctgccctaaa

VHL-UTR-mut2f: p-acatgccgtttgaCACACACgtttttggtgttg

VHL-UTR-mut2r: p-tttttttttgtttttttggtttctttttg

VHL siRNA and anti-*miR-101* oligos were purchased from Shanghai GenePharma.

### Quantitative real time-polymerase chain reactions (qRT-PCR)

Total RNA was isolated using TRIzol reagent (Invitrogen). RNA was reverse-transcribed by RevertAidFirst Strand cDNA Synthesis Kit (Thermo Scientific) according to the manufacturer’s instructions. Quantitative real-time PCR was performed using SYBR Green PCR real-time PCR Master MIX (Toyobo). miRNA was isolated using mirVana™ miRNA Isolation Kit(Life Technologies). The TaqMan® microRNA reverse transcription kit (Applied Biosystems) was used to synthesize the cDNA for *miR-101*. The expression level of U6 snRNA was used as an internal control for normalization.

### Cell proliferation assay

20000/well cells were seeded into a six-well plate and maintained in Dulbecco’s Modified Eagle’s Medium (DMEM) with 10% fetal bovine serum (FBS), changing half of the medium every other day. Then counting cell number at indicated time points.

### Luciferase assay

The dual-luciferase assay was performed 48 h after transfection according to the manufacturer’s instructions (Promega, Madison, WI, USA). The Renilla luciferase was used as a control reporter. The ratio of Renilla luciferase to firefly luciferase was normalized to the negative control. Three independent experiments were performed.

### Apoptosis assay

Cells were harvested and washed twice with pre-cooled PBS and then were incubated in the binding buffer with a mixture containing annexin V (Biolegend) and propidium iodide (Sungene Biotech ) for 15 min in darkness. Cells were measured by flow cytometry. Three independent experiments were performed.

### Cell cycle assay

The cells were fixed in chilled 75% ethanol and stained with a propidium iodine (PI) solution contains 100 ug/ml RNase (Tiangen Biotech) and 50 ug/ml PI (Biolegend) in PBS for cell cycle analysis.

### Colony formation assay

1000/well cells were seeded into a six-well plate and maintained in Dulbecco’s Modified Eagle’s Medium (DMEM) containing 10% fetal bovine serum (FBS) for 14 days, changing half of the medium every other day. Colonies were fixed in 4% paraformaldehyde for 20 min at room temperature, and then stained with crystal violet for 30 min at room temperature. The numbers of colonies were counted and pictures were captured.

### Western blot

Cells were collected and lysed in Radio-Immunoprecipitation Assay (RIPA) buffer (Thermo Fisher, Rockford, IL, USA) containing 0.02% complete Protease Inhibitor EASY packs EDTA-Free (Roche Applied Science). Then the protein was separated using 12% polyacrylamide gel, and transferred to polyvinyl difluoride membranes. After blocking with 5% bovine serum albumin (BSA) in TBS/Tween20 (TBST) and incubated with specific primary antibodies at 4 °C overnight, followed by a 5 min wash with TBS-T, which was repeated three times. After this, the membranes were incubated with horseradish peroxidase conjugated anti-mouse or anti-rabbit IgG secondary antibody (Santa Cruz Biotechnology, Santa Cruz, CA, USA) at room temperature for 1 h, followed a 5 min wash with TBST, which was repeated three times. Then the membranes were analyzed with the ECL chemiluminescent detection system (Bio-Rad).

The following antibodies were used in this study: anti-actin monoclonal (Santa Cruz Biotechnology, Santa Cruz, CA, USA) , anti-HIF1α polyclonal (Cell Signaling Technology), anti-VEGFA polyclonal, anti-cyclinD1 polyclonal (ProteinTech Group, Chicago, IL, USA) and anti-VHL polyclonal (Cell Signaling Technology).

### EdU (5-ethynyl-2’-deoxyuridine) incorporation assay

Cells were seeded on cover slips for 48 h. The assay was performed with EdU cell proliferation assay kit (Thermo Fisher, Rockford, IL, USA) according to the manufacturer’s instructions. Briefly, EdU was added to the culture media for 2 h and the final concentration was 10 μM. After labeling, cells were fixed in 4% formaldehyde. Then stained cells with Edu and counterstained with Hoechest, mounted in standard mounting media and imaged by fluorescence microscopy.

## Additional Information

**How to cite this article**: Liu, N. *et al.* *MicroRNA-101* targets von Hippel-Lindau tumor suppressor (*VHL*) to induce HIF1α mediated apoptosis and cell cycle arrest in normoxia condition. *Sci. Rep.* **6**, 20489; doi: 10.1038/srep20489 (2016).

## Supplementary Material

Supplementary Information
