# Supplementary material for: MicroRNA-101 targets von Hippel-Lindau tumor suppressor (VHL) to induce HIF1α mediated apoptosis and cell cycle arrest in normoxia condition
Source: Sci Rep. 2016 Feb 4;6:20489. doi: 10.1038/srep20489 (PMC4740907; doi:10.1038/srep20489)
Supplement: Supplementary Information [file srep20489-s1.pdf]

MicroRNA-101 targets von Hippel-Lindau tumor suppressor (*VHL*) to induce HIF1α mediated apoptosis and cell cycle arrest in normoxia condition

Ning Liu[1], Wu-Yan Xia[1], Shan-Shan Liu[1], Hai-Yan Chen[1], Lei Sun[1], Meng-Yao Liu[1], Lin-Feng Li[1], Hong-Min Lu[1], Yu-Jie Fu[1], Pei Wang[1], Hailong Wu[1†], Jian-Xin Gao[1†]

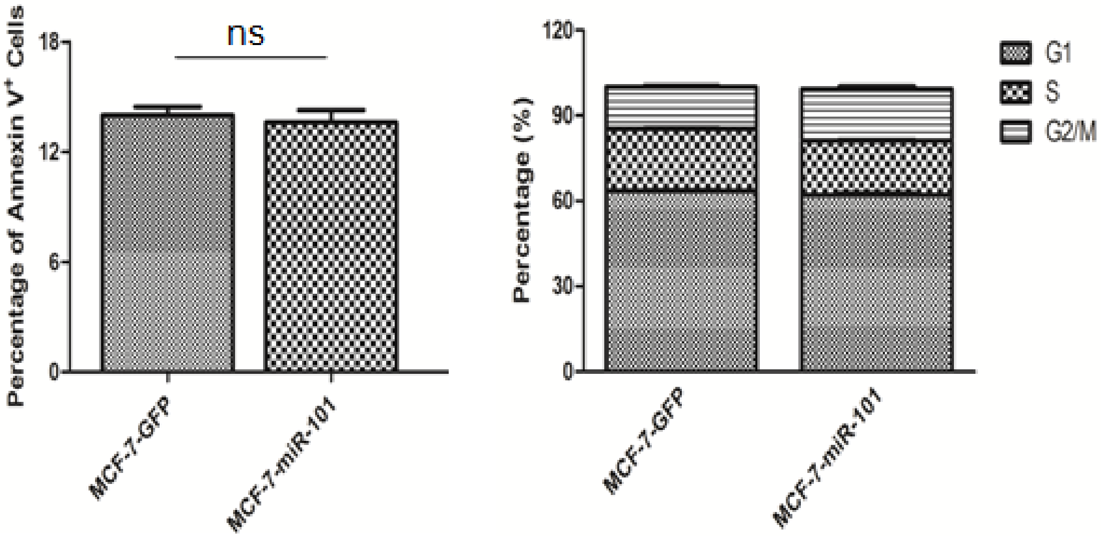

Figure S1. *miR-101* has no effect on apoptosis and cell cycle in MCF-7 cells under hypoxia exposure.
Apoptosis and cell cycle assays in MCF-7-miR-101 under hypoxia exposure.

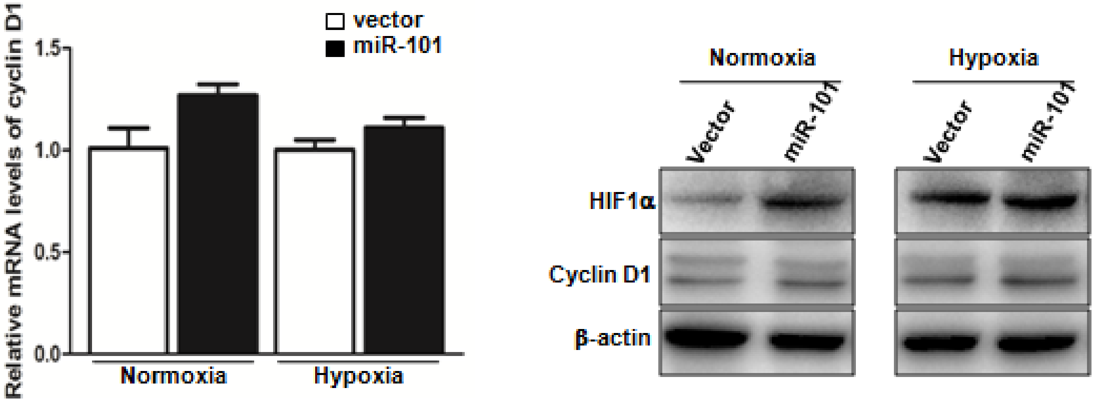

Figure S2. miR-101 does not affect cyclinD1 levels in either normoxia or hypoxia condition.
mRNA and protein levels of CyclinD1 in MCF-7 cells with *miR-101* expression detected by qPCR and western respectively under normoxia or hypoxia condition.

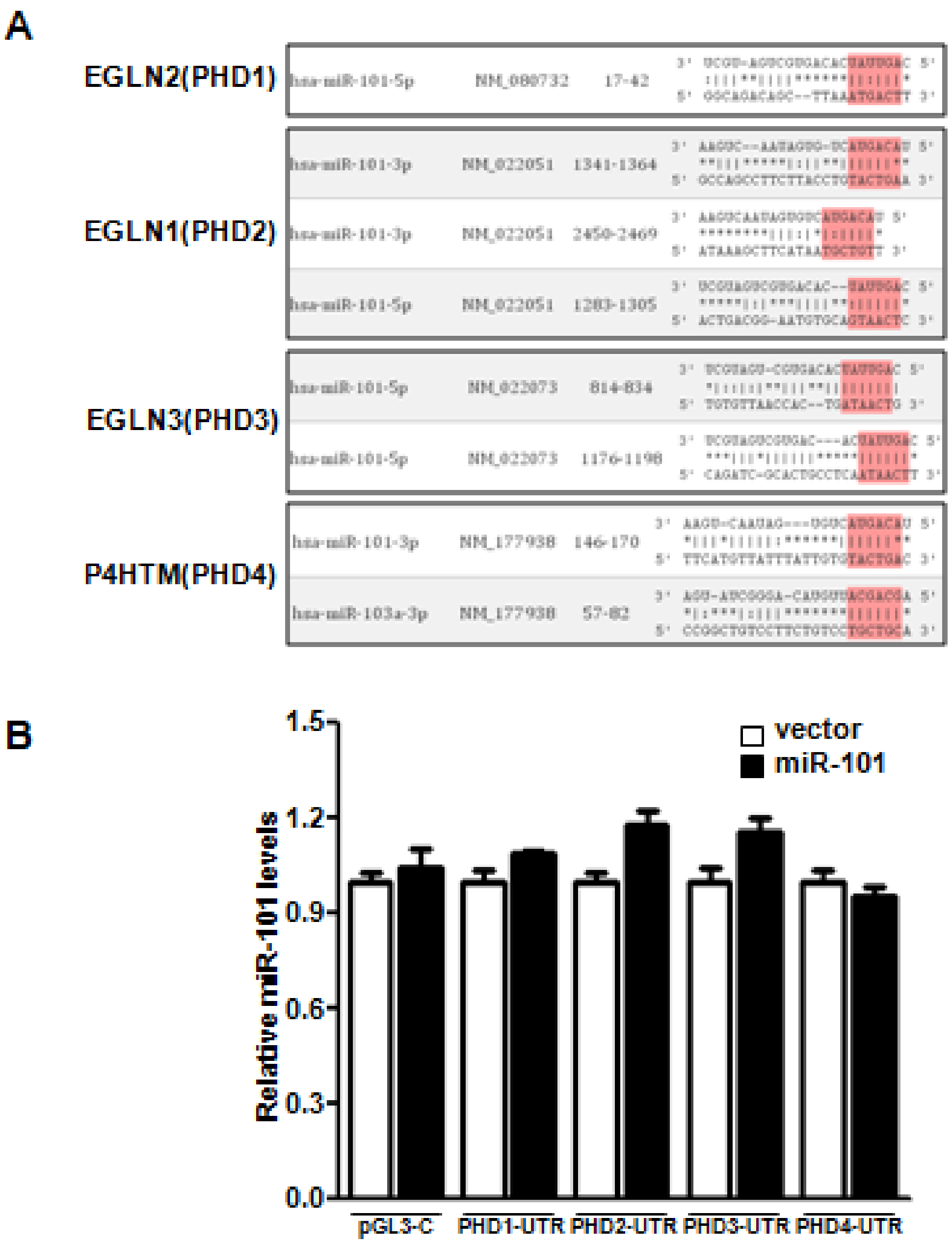

Figure S3. PHD1-4 are not the direct target of *miR-101*.

(A) Predicted binding sites of miR-101 on the 3'UTR regions of PHD1-4 by FINDTAR3. (B) Effect of *miR-101* on the luciferase activity of pGL3-control, PHD1-UTR, PHD2-UTR, PHD3-UTR and PHD4-UTR.
